# Effects of dietary protein levels on growth, carcass characteristics, meat quality, and hematological parameters of BAU black and white duck of Bangladesh

**DOI:** 10.5455/javar.2026.m1060

**Published:** 2026-06-22

**Authors:** Maksuda Aktar, Krishna Chandra Barman, Pranto Saha, Abu Jofar Mohammad Ferdaus, Mohammad Alam Miah, Mohammad Shamsul Alam Bhuiyan

**Affiliations:** 1 Department of Animal Breeding and Genetics, Faculty of Animal Husbandry, Bangladesh Agricultural University, Mymensingh, Bangladesh; 2 Department of Livestock Services, Ministry of Fisheries and Livestock, Dhaka, Bangladesh; 3 Department of Physiology, Faculty of Veterinary Science, Bangladesh Agricultural University, Mymensingh, Bangladesh

**Keywords:** Bangladesh, duck, meat, protein, ration

## Abstract

**Objectives:** Protein is a vital element in poultry diets for growth, muscle and feather development, and overall health. This study aimed to optimize the amount of crude protein (CP) in the ration of BAU Black and White crossbred ducks for better growth, carcass characteristics, and meat quality.

**Materials and Methods:** A total of 264-day-old ducklings were randomly and equally distributed into four treatments allocating 16.50% (T_1_), 18.00% (T_2_), 19.50% (T_3_) and 21.00% (T_4_) CP in the ration. Live weight and feed intake data were measured weekly. At 10 weeks of age, 24 blood samples were collected from 4 treatments for investigating hematological parameters, and those ducks were slaughtered for analysis of carcass characteristics and meat quality.

**Results:** Body weight differed significantly (*p* < 0.05) from the 3^rd^ to 10^th^ weeks, and at the 4^th^, 6^th^, 8^th^, and 10^th^ weeks of age, there were significant (*p* < 0.01) differences in feed conversion ratio (FCR) and average daily gain (ADG) among the treatments. T_3_ (19.50%) showed the highest live weight, ADG, and better FCR among the treatments. Carcass traits such as carcass weight, breast meat weight, giblet weight, and head weight showed significantly better in Treatment T_3_. Meat quality analysis depicted significant differences (*p* < 0.05) in drip loss and cooking loss parameters only in the breast meat, while lightness attributes (L*) of meat color differed significantly (*p* < 0.05) for thigh muscles. Besides, among the investigated hematological parameters, only total erythrocyte count (TEC) varied significantly (*p* < 0.01).

**Conclusions:** This study confirmed a 19.50% dietary CP level for optimum growth, carcass yield, and meat quality that could be utilized in the rearing of BAU Black and White ducks.

## 1. Introduction

Ducks represent the second-largest source of poultry meat and egg production worldwide after chickens. Duck husbandry in Bangladesh is mostly scavenging and semi-intensive in nature and predominantly managed by smallholder farmers. Ducks are one of the valuable genetic resources, particularly for rural women’s income generation and their livelihood component. In Bangladesh, about one-ninth of total land is low-lying and marshy, which is suitable for raising ducks [1]. They are valued for their better disease resistance, longer productive life, and outstanding foraging skills [2]. Duck meat has gained popularity among all Bangladeshi communities, regardless of caste, religion, or creed. However, the growing demand of the duck meat requirement has been fulfilled by the slow-growing indigenous or a few egg-type exotic duck breeds, such as Indian Runner, Jinding, and Khaki Campbell. Therefore, it is highly justified to select a duck genotype having better growth performance and adaptability under traditional management practices of Bangladesh for fulfilling the country’s demand.

Crossbreeding or upgrading has been practiced with many duck populations globally to harness hybrid vigor in the offspring [3, 4]. Using more productive exotic and better-suited local duck breeds could be a viable option for developing crossbred or upgraded duck genotypes suitable for the agroclimatic conditions of Bangladesh. Recently, a crossbred duck named BAU Black and White has been developed through reciprocal crossing between exotic Pekin and indigenous Nageswari duck breeds. This crossbred genotype reveals the potential for increased meat and egg production over the average of the parental genotypes [5]. The morphology, growth performance, and carcass characteristics of BAU Black and White crossbred ducks have been investigated in previous research [3, 5], but the optimum dietary protein requirement for this crossbred has not yet been optimized.

Duck rations must provide all essential nutrients for maintenance, growth, and reproduction, whether scavenged or fully supplied. Crude protein content in a ration is a prime concern for supplying all essential amino acids and is one of the main factors determining the amount duck diets cost [6, 7]. Nitrogen and ammonia emissions from poultry waste are major pollutants, influenced by dietary protein levels, which also increase feed costs [8]. Growth performance is significantly influenced by dietary crude protein levels, and feeding birds with optimum dietary crude protein improved their feed efficiency and average daily gain [6, 9]. However, either an excess or a lack of dietary protein in the diet can cause metabolic diseases that affect growth performance, interact with physiological functions, and inhibit nutrition metabolism [10, 11]. According to Zeng et al. [12], feeding 19.00% crude protein for 15–35 days is appropriate for optimizing growth performance and carcass characteristics in Pekin ducks, which also minimizes crude protein loss. Crude protein requirements differ from duck population to population and are influenced by genotype, age of the bird, type of duck, and management practices [13].

However, there is limited literature available on crude protein optimization in ducks, and it is absent for BAU Black and White crossbreds. Thus, this research investigated the impact of dietary crude protein levels on growth performance, carcass characteristics, meat quality, and hematological parameters of BAU Black and White crossbred ducks, and an optimal protein level could be recommended for improved productivity of this crossbred genotype.

## 2. Materials and Methods

### 2.1. Ethical approval

This study was conducted following guidelines set by the Ethical Standard of Research Committee, Bangladesh Agricultural University Research System (BAURES) (Approval No.: BAURES/ESRC/103/AH./2025).

### 2.2. Experimental design

A total of 264-day-old ducklings were hatched from the 5^th^ generation flock of BAU Black and White crossbred ducks that have been maintained at Bangladesh Agricultural University (BAU) Artificial Insemination Center, Mymensingh, Bangladesh, and distributed equally into four groups for this experiment. The ducklings were given a commercial starter ration (Nourish Feeds Ltd.) during the first week. The experimental birds received dose-response rations with 4 dietary crude protein (CP) levels (16.50%, 18.00%, 19.50%, and 21.00%) from the 2^nd^ week to the 10^th^ week of age. These levels corroborate 4 dietary treatments (T_1_, T_2_, T_3_, and T_4_) with similar metabolic energy contents (ME: 2850 kcal/kg). The birds’ dietary requirements were fixed according to the basis of dual-purpose ducks. Table 1 represents the experimental composition of the ration and analyzed value of CP content in each ration.

**Table 1.** Composition of experimental rations.

Feed ingredient	Dietary protein level			
	T_1_	T_2_	T_3_	T_4_
Maize (kg)	53.60	50.00	47.00	44.50
Rice polish (kg)	21.10	25.00	25.00	25.00
Soyabean meal (kg)	17.00	18.00	20.00	20.15
Protein Concentrate (kg)	1.90	4.30	6.00	8.80
Lime stone (kg)	1.83	1.80	1.50	1.20
Di-calcium phosphate (kg)	0.80	0.37	0.10	-
Vitamin-mineral premix (kg)^*^	0.25	0.25	0.25	0.25
Salt (kg)	0.20	0.20	0.20	0.20
DL-methionine (kg)	0.07	0.05	-	-
Lysine (kg)	0.20	0.50	-	-
Calculated composition				
Metabolizable energy (kcal/kg)	2851	2852	2852	2854
Crude protein (%)	16.50	18.00	19.53	21.00
Crude fiber (%)	4.37	4.44	4.48	4.47
Crude fat (%)	6.31	6.51	6.55	6.67
Lysine (%)	1.00	1.01	1.09	1.20
Methionine (%)	0.40	0.42	0.41	0.46
Calcium (%)	1.09	1.13	1.06	1.10
Phosphorus (%)	0.40	0.41	0.41	0.48
Analyzed composition				
Crude protein (%)	16.54	17.98	19.52	21.32
Ca%	1.32	1.16	1.18	1.19
P%	0.56	0.50	0.53	0.51
Fat%	3.79	3.85	4.52	3.52

^*^Constituents of the provided vitamin-mineral premix are fat-soluble vitamins (A, D_3_, E, K); water-soluble B vitamins (B_1_, B_2_, B_3_, B_12_, niacin, biotin, folic acid); and trace minerals (iron, copper, zinc, manganese, iodine, selenium, cobalt).

### 2.3. Husbandry practices

The ducklings were raised on a concrete floor with bedding made of rice husk. Mulching was practiced every other day in the second half of the experiment to keep the litter dry. Brooding was provided until the chicks reached three weeks of age. Birds were allowed to move daily in the run for a period of 3–4 h. Handmade wet mash feed was provided twice a day, in the morning and afternoon, based on weekly feed requirements. At the same time, plastic drinkers were provided proportional access to safe drinking water. Ducklings received vaccinations against avian influenza, duck cholera, and duck plague following the guidelines of Ahmad et al. [3].

### 2.4. Growth performance

The live weight of the duck was measured weekly in the morning before offering them feed and water. Any leftover feed was measured regularly in the following morning to estimate feed consumption and was continued until the 10^th^ week of age. Average daily gain (ADG) and feed conversion ratio (FCR) were determined using information on body weight and feed intake. During the experimental period, the death record showed that only 5 birds (1.89% of the total population) died during the first 2 weeks of the trial.

### 2.5. Blood sampling and hematological analysis

Six ducks (3 males and 3 females) were chosen randomly from each treatment at the end of the 10^th^ week of age, and a total of 24 blood samples (~3 ml) were collected from the wing vein using an EDTA-coated tube for hematological tests. Hematological parameters such as hemoglobin (Hb), total erythrocyte count (TEC), erythrocyte sedimentation rate (ESR), and packed cell volume (PCV) were measured by using the automated hematology analyzer Sysmex XN-2000 (Sysmex, Kobe, Japan).

### 2.6. Carcasses and meat quality parameters

A total of 24 ducks, those selected for blood sampling, were slaughtered by the halal method at the 10^th^ week of age; then they were thoroughly bled out and given a warm water bath for defeathering. Finally, the eviscerated carcasses were suspended for a while and wiped with kitchen tissue to eliminate water from the body and were measured by a digital weighing scale. The different body parts were sectioned according to the method outlined by Ahmad et al. [3]. The investigated carcass traits were body weight (BWT), carcass weight (CWT), dressing percentage (DP%), back-half weight (BHWT), breast meat weight (BMWT), thigh with drumstick weight (TWDT), wing weight (WWT), neck weight (NWT), giblet weight (GWT), and head weight (HWT).

Skinning and deboning the breast and thigh muscles from both sides of the carcasses allowed for the investigation of meat quality parameters such as water holding capacity (WHC), cooking loss (CL), drip loss (DL), and meat color (L*, lightness; a, redness; and b, yellowness). For this, 10 gm of each meat sample were wrapped in heat-resistant foil paper, placed in a water bath at 80°C for half an hour, dried, and weighed in order to calculate CL. The percentage of the cooked sample that was lost was used to compute CL [14].


\[
{\rm CL}\,\left(\%\right)\;=\;\frac{{{\rm W}1-{\rm W}2}}{{{\rm W}1}}\; \times \;100
\]

Where W1 and W2 were meat weight before and after cooking, respectively.

In order to measure DL, 15 gm samples of meat were placed in inflated polythene bags, hung on a string in an airtight box, and then stored at 4°C for 24 h. DL was then computed according to the method outlined by Berri et al. [15].


\[
{\rm Drip\,loss}\,\left(\% \right)\;=\;\;\frac{{{\rm Sample\,initial\,weight}-{\rm sample\,weight\,after}\,24{\rm h}}}{{{\rm Sample\,initial\,weight}}}\;\times\;100
\]

The centrifugation assay was used to measure the WHC. 1 gm of the sample was minced into tiny cubes and put in a microcentrifuge tube to be centrifuged at 4°C for 10 min with 1000 RCF. The WHC was calculated by the following formula:


\[
{\rm WHC}\,\left(\% \right)\;=\;100\;-\;\left({\frac{{{\rm Sample\,weight\,before\,centrifugation}-{\rm Sample\,weight\,after\,centrifugation}}}{{{\rm Sample\,weight\,before\,centrifugation}}}\; \times \;100} \right)
\]

The DP% was calculated by the following formula:


\[
{\rm DP}\,\% \;=\;\frac{{{\rm Whole\,carcass\,weight}}}{{{\rm Live\,weight}}}\; \times \;100
\]

The color profile (L*, a, and b) of the meat of the breast and thigh was measured at three points on the dorsal surface of each sample after 30 min of exposure to ambient air using a colorimeter (CR-400/410, Konica, Japan).

### 2.7. Data analysis

The data from the experimental record books was compiled into an Excel sheet. After removing extreme values, descriptive statistics were computed, including the mean, standard error, frequency, and percentage distribution. The Agricolae package in R was used to conduct ANOVA with a completely randomized design [16], and the Pastecs program was used to check for significant mean differences [17]. The impact of dietary crude protein percentages was calculated using the following model, where CP content was considered as a fixed effect.


\[
{{\rm Y}_{{\rm ij}}}\;=\;\;{\rm \mu} + \;{{\rm T}_{\rm i}}\; + \;{{\rm e}_{{\rm ij}}}
\]

Y_ij_ = the dependent variable (traits)

μ = the overall mean

T_i_ = the fixed effect of the i^th^ treatments (T_1_, T_2_, T3, and T_4_)

e_ij_ = the residual error

## 3. Results

### 3.1. Performance of growth

The effects of dietary CP percentage on the growth performance of BAU Black and White crossbred ducks up to the 10^th^ week of age are shown in Table 2. Growth performance differed significantly across the treatments except for day-old weight (DOW) to 2^nd^ week. However, weekly growth performance did not rise linearly with progression of age (Table 2). At the 3rd week of age, ducks in T_4_ (584.67 ± 10.05 gm) attained the highest body weight, whereas T_1_ (548.18 ± 8.89 gm) recorded the lowest (*p* < 0.05). Treatments T_2_ (577.03 ± 9.82 gm) and T_3_ (576.22 ± 9.08 gm) showed intermediate values, which were statistically similar to T_4_ but higher than T_1_. Treatment T_1_ (16.50%) had the lowest (*p* < 0.01) BWT at the 10^th^ week of age; the ducks fed a 19.50% (T_3_) ration had the highest (*p* < 0.01) BWT and differed insignificantly from the treatments T_4_ (21.00%) and T_2_ (18.00%). There were highly significant (*p* < 0.01) variations observed among the treatment groups for FCR and ADG except for the 1^st^ and 2^nd^ weeks of age (Table 3). During the first few weeks, better FCR was found across the treatment groups; however, it declined gradually with the progression of age.

**Table 2.** Effect of crude dietary protein percentage on growth performance of BAU Black and white crossbred ducks up to the 10^th^ week of age.

Age in Week	Body weight^1^				*p*-value^2^
	T_1_ (n = 66)	T_2_ (n = 66)	T_3_ (n = 66)	T_4_ (n = 66)	
DOW	43.46 ± 0.72 (66)	43.55 ± 0.75 (66)	45.23 ± 0.61 (66)	43.79 ± 0.70 (66)	0.237
1^st^	148.77 ± 1.98 (65)	148.92 ± 2.08 (64)	146.42 ± 2.05 (62)	149.76 ± 2.21 (62)	0.706
2^nd^	304.56 ± 4.57 (62)	303.30 ± 4.69 (63)	306.25 ± 5.15 (60)	303.85 ± 4.61 (66)	0.975
3^rd^	548.18^b^ ± 8.89 (66)	577.03^ab^ ± 9.82 (64)	576.22^ab^ ± 9.08 (63)	584.67^a^ ± 10.05 (61)	0.034
4^th^	811.52^a^ ± 12.89 (65)	817.02^a^ ± 12.57 (63)	814.67^a^ ± 12.50 (61)	759.16^b^ ± 11.94 (63)	0.002
5^th^	966.94^ab^ ± 14.41 (65)	971.29^a^ ± 15.01 (65)	974.27^a^ ± 14.89 (63)	917.88^b^ ± 14.03 (65)	0.019
6^th^	1197.50^ab^ ± 16.15 (64)	1197.75^ab^ ± 17.70 (64)	1209.44^a^ ± 17.23 (64)	1137.29^b^ ± 17.44 (63)	0.014
7^th^	1397.81^ab^ ± 18.92 (64)	1368.42^ab^ ± 18.92 (65)	1417.23^a^ ± 20.44 (64)	1328.36^b^ ± 20.02 (62)	0.010
8^th^	1565.56^ab^ ± 21.49 (63)	1553.73^ab^ ± 21.90 (66)	1618.67^a^ ± 21.46 (63)	1504.19^b^ ± 22.72 (64)	0.003
9^th^	1700.91^b^ ± 22.13 (63)	1705.03^ab^ ± 22.98 (66)	1788.05^a^ ± 22.68 (64)	1687.27^b^ ± 24.68 (64)	0.009
10^th^	1795.59^b^ ± 21.66 (59)	1824.45^ab^ ± 21.93 (65)	1886.86^a^ ± 23.24 (62)	1806.71^ab^ ± 24.73 (61)	0.026

^1^ T_1_, T_2_, T_3_, and T_4_ represent different crude protein levels in the duck rations as 16.50%, 18.00%, 19.50%, and 21.00%, respectively. ^2^ means different superscripts in the same row differ significantly in the protein content level (*p* < 0.05).

**Table 3.** Effect of dietary crude protein percentage on average daily gain (gm) and feed conversion ratio in BAU Black and white crossbred ducks up to the 10^th^ week of age.

Age in weeks	Trait^1^	Treatment^2^				*p*–Value^3^
		T_1_ (n = 66)	T_2_ (n = 66)	T_3_ (n = 66)	T_4_ (n = 66)	
1^st^	ADG	14.81 ± 0.16	14.91 ± 0.17	14.49 ± 0.18	15.14 ± 0.17	0.071
	FCR	0.92 ± 0.01	0.92 ± 0.01	0.95 ± 0.01	0.91 ± 0.01	0.079
2^nd^	ADG	22.33 ± 0.33	21.85 ± 0.36	22.77 ± 0.41	21.51 ± 0.31	0.069
	FCR	1.99 ± 0.02	2.03 ± 0.03	1.94 ± 0.03	2.03 ± 0.02	0.077
4^th^	ADG	37.28^a^ ± 0.59	34.44^b^ ± 0.46	34.31^b^ ± 0.52	24.30^c^ ± 0.26	0.000
	FCR	2.07^c^ ± 0.03	2.23^b^ ± 0.03	2.24^b^ ± 0.04	3.13^a^ ± 0.03	0.000
6^th^	ADG	33.12^a^ ± 0.38	32.21^ab^ ± 0.48	33.29^a^ ± 0.33	31.21^b^ ± 0.47	0.001
	FCR	2.86^b^ ± 0.03	2.92^ab^ ± 0.04	2.83^b^ ± 0.03	3.03^a^ ± 0.04	0.001
8^th^	ADG	24.06^b^ ± 0.48	25.10^b^ ± 0.44	28.90^a^ ± 0.42	24.01^b^ ± 0.31	0.000
	FCR	3.92^bc^ ± 0.09	4.13^ab^ ± 0.03	3.79^c^ ± 0.06	4.27^a^ ± 0.04	0.000
10^th^	ADG	16.60^c^ ± 0.38	17.89^b^ ± 0.29	15.66^c^ ± 0.27	19.44^a^ ± 0.34	0.000
	FCR	6.62^ab^ ± 0.16	6.76^a^ ± 0.09	6.95^a^ ± 0.16	6.26^b^ ± 0.10	0.001

^1^ ADG, average daily gain; FCR, feed conversion ratio. ^2^ T_1_, T_2_, T_3_, and T_4_ represent different crude protein levels in the duck rations: 16.50%, 18.00%, 19.50%, and 21.00%, respectively. ^3^ means with different superscripts in the same row differ significantly in the protein content level (*p* < 0.05).

### 3.2. Carcass characteristics

The effect of dietary CP levels on carcass characteristics at the tenth week of age is shown in Table 4. Slaughtered ducks showed significant differences (*p* < 0.05) in BWT, CWT, BMWT, GWT, and HWT among the different treatment groups, while other carcass traits remained unaffected (*p* > 0.05). Birds fed with a 19.50% CP-containing ration (T_3_) had significantly higher BWT, CWT, BMWT, and HWT compared to the treatments T_1_ and were insignificantly different from T_4_ and T_2_. Further, the highest GWT found in T_3_ and T_1_, insignificantly different from T_2_ and T_4_, showed the lowest weight.

**Table 4.** Effect of crude dietary protein levels on carcass characteristics of BAU Black and White crossbred ducks at the 10^th^ week of age.

Trait^1^	Carcass characteristics^2^				*p*-value^3^
	T_1_ (n = 6)	T_2_ (n = 6)	T_3_ (n = 6)	T_4_ (n = 6)	
BWT	1849.75^b^ ± 78.77	1990.50^ab^ ± 48.86	2106.80^a^ ± 29.10	1950.50^ab^ ± 70.50	0.041
CWT	1367.50^b^ ± 57.00	1458.40^ab^ ± 55.71	1579.20^a^ ± 39.93	1442.25^ab^ ± 25.15	0.044
DP%	75.52 ± 1.18	73.65 ± 1.90	74.96 ± 1.62	74.37 ± 1.04	0.819
BHWT	408.75 ± 27.85	454.25 ± 17.90	469.67 ± 17.94	433.20 ± 29.94	0.337
BMWT	286.00^b^ ± 19.04	340.75^ab^ ± 7.22	365.33^a^ ± 10.11	346.75^ab^ ± 32.73	0.043
TWD	240.80 ± 20.49	246.80 ± 5.79	260.17 ± 9.18	234.25 ± 32.38	0.744
WWT	182.80 ± 10.37	193.83 ± 6.18	202.83 ± 7.70	194.50 ± 9.83	0.474
NWT	199.75 ± 13.89	222.40 ± 4.46	232.80 ± 11.66	203.25 ± 4.82	0.085
GWT	101.75^a^ ± 3.52	118.83^ab^ ± 5.29	135.20^a^ ± 4.44	115.60^b^ ± 4.96	0.003
HWT	91.25^b^ ± 3.35	100.40^ab^ ± 3.11	105.60^a^ ± 3.59	103.00^ab^ ± 3.56	0.048

^1^ BWT, body weight; CWT, carcass weight; DP, dressing percentage; BHWT, back-half weight; BMWT, breast meat weight; TWD, thigh with drumstick; WWT, wing weight; NWT, neck weight; GWT, giblet weight; HWT, head weight. ^2^ T_1_, T_2_, T_3,_ and T_4_ represent different crude protein levels in the duck rations as 16.50%, 18.00%, 19.50%, and 21.00%, respectively. ^3^ means with different superscripts in the same row differ significantly in the protein content level (*p* < 0.05).

### 3.3. Meat quality parameters

The influence of dietary protein percentage on meat quality parameters is shown in Table 5. Significant differences (*p* < 0.05) were found among the four dietary treatment groups for DL and CL attributes in breast meat muscle. The DL of breast muscle was substantially (*p* < 0.05) higher in ducks fed a 19.50% (T_3_) ration and differed insignificantly from T_4_ and T_1_, while the lowest value was found in treatment T_2_. Further, T_3_ and T_2_ had significantly higher (*p* < 0.05) CL of breast muscle at the 10^th^ week compared to T_4_ and T_1_ treatments. However, remaining meat quality parameters did not differ significantly (*p* > 0.05) among the treatments.

**Table 5.** Effect of dietary crude protein levels on meat quality parameters of BAU Black and White crossbred ducks at the 10^th^ week of age.

Treatment^2^	Meat quality paramter^1^						
	Breast meat				Thigh meat		
	DL%	CL%	WHC%		DL%	CL%	WHC%
T_1_ (n = 6)	2.88^ab^ ± 0.15	34.42^b^ ± 1.06	94.06 ± 1.04		1.17 ± 0.14	34.33 ± 1.64	97.35 ± 0.38
T_2_ (n = 6)	2.14^b^ ± 0.37	37.41^a^ ± 0.80	92.97 ± 0.39		0.92 ± 0.09	37.34 ± 0.38	97.15 ± 0.32
T_3_ (n = 6)	3.63^a^ ± 0.21	37.78^a^ ± 0.97	91.97 ± 0.84		1.10 ± 0.19	34.06 ± 1.21	93.58 ± 3.05
T_4_ (n = 6)	3.22^ab^ ± 0.38	35.22^b^ ± 0.74	94.59 ± 1.00		1.09 ± 0.17	34.56 ± 1.16	96.31 ± 0.55
*p* - value^3^	0.019	0.044	0.182		0.691	0.374	0.275

^1^ DL, drip loss; CL, cooking loss; WHC, water holding capacity. ^2^ T_1_, T_2_, T_3_, and T_4_ represent different crude protein levels in the duck rations as 16.50%, 18.00%, 19.50%, and 21.00%, respectively. ^3^ means with different superscripts in the same row differ significantly in the protein content level (*p* < 0.05).

Dietary CP levels had a significant effect (*p* < 0.05) on only the lightness (L*) of thigh meat, while the redness (a) and yellowness (b) of meat varied insignificantly among the different dietary CP levels (Table 6). However, when dietary CP levels increased, the L* decreased. More particularly, T_2_ showed the highest lightness that differed insignificantly with T_1_ and T_3_, and the lowest value was found in the treatment T_1_.

**Table 6.** Effect of dietary crude protein levels on meat color attributes at 10^th^ week of age.

Treatment^2^	Meat color^1^							
	L*			a			b	
	BM	TM		BM	TM		BM	TM
T_1_ (n = 6)	38.51 ± 5.41	31.91^cd^ ± 5.19		9.09 ± 0.99	10.28 ± 1.74		3.40 ± 0.51	5.46 ± 1.13
T_2_ (n = 6)	41.35 ± 5.61	35.80^c^ ± 3.80		11.40 ± 0.81	10.67 ± 0.96		6.35 ± 1.79	5.67 ± 1.83
T_3_ (n = 6)	35.21 ± 2.79	24.26^cd^ ± 4.33		9.08 ± 0.90	11.36 ± 2.02		4.34 ± 1.47	4.25 ± 1.13
T_4_ (n = 6)	27.11 ± 1.40	15.23^d^ ± 1.52		8.44 ± 1.09	7.35 ± 1.17		1.94 ± 0.67	3.27 ± 0.55
*p-*value^3^	0.147	0.011		0.190	0.333		0.135	0.457

^1^ L*, Lightness; a, Redness; b, Yellowness; BM, breast meat, TM, thigh meat. ^2^ T_1_, T_2_, T_3_, and T_4_ represent different crude protein levels in the duck rations as 16.50%, 18.00%, 19.50%, and 21.00%, respectively. ^3^ means with different superscripts in the same row differ significantly in the protein content level (*p* < 0.05).

### 3.4. Hematological parameters

Significant (*p* < 0.01) increases in TEC levels were observed in the experiment across various dietary CP percentages (Table 7). However, there was no statistical difference in HB, ESR, and PCV among the different dietary protein percentages.

**Table 7.** Effect of dietary crude protein levels on hematological parameters of BAU Black and White crossbred ducks at the 10^th^ week of age.

Hematological parameter^1^	Treatment^2^				*p*-value^3^
	T_1_ (n = 6)	T_2_ (n = 6)	T_3_ (n = 6)	T_4_ (n = 6)	
Hb (gm/dl)	11.73 ± 0.72	12.09 ± 0.45	11.65 ± 0.69	12.39 ± 0.37	0.773
TEC (million/mm^3^)	2.92^ab^ ± 0.19	3.53^a^ ± 0.20	2.71^b^ ± 0.11	3.30^ab^ ± 0.11	0.006
ESR (mm/1^st^ h)	2.00 ± 0.63	2.43 ± 0.61	2.14 ± 0.63	1.50 ± 0.22	0.713
PCV (%)	39.57 ± 2.42	38.57 ± 2.13	40.00 ± 2.40	41.43 ± 2.19	0.847

^1^ Hb, hemoglobin; TEC, total erythrocyte count; ESR, erythrocyte sedimentation rate; PCV, packed cell volume. ^2^ T_1_, T_2_, T_3_, and T_4_ represent different crude protein levels in the duck rations as 16.50%, 18.00%, 19.50%, and 21.00%, respectively. ^3^ means with different superscripts in the same row differ significantly in the protein content level (*p* < 0.05).

## 4. Discussion

Dietary protein is the costliest component of a ration, and thus, attention has to be paid for formulating a balanced and cost-effective ration. This critical nutrient directly influences the reproductive and productive performance of flocks for producing meat or eggs [18]. Therefore, determining the optimum CP requirements of the recently developed BAU Black and White crossbred ducks is essential for their optimum productivity under local production conditions. In the present study, variations in dietary CP levels influenced growth performance, including BWT, ADG, and FCR.

Previous studies on Pekin ducks fed with different CP percentages at an isoenergic level have demonstrated higher body weights than those observed in the present findings, likely due to differences in genotype, dietary composition, and management system [12, 19]. Similarly, Wang et al. [20] reported that Pekin ducks receiving 16.5% CP achieved an average body weight of 3512 ± 54.02 gm up to 49 days, which was higher than the weights recorded in this study. According to Joshi et al*.* [21], the Khaki Campbell ducks fed with 18%, 20%, and 22% CP produced lower average body weights compared to the current study, where genetic differences appear to be the major contributing factor. Baeza et al. [22] reported the optimal CP levels in the starter (0 to 3 weeks), grower (4 to 7 weeks), and finisher (8 to 10 weeks) rations of mule ducks as 23.5%, 15.4%, and 13.8%, respectively. They found greater body weights up to the 10^th^ week than those recorded in the current study, again highlighting the influence of genetic potential and dietary variation. Conversely, as reported by Ebnat et al. [5], the body weight of Pekin × Nageswari crossbred ducks fed diets containing a 16.50% CP level was lower than in the current study, possibly due to variations in the CP content of grower ration. The lower body weight observed in this study for higher dietary protein (T_4_) compared to other protein levels can be due to inefficiencies associated with excessive dietary protein, including imbalanced protein-to-energy ratios, increased nitrogen excretion, and higher metabolic costs, which is similar to Yu et al. [23].

Body weight gain and FCR are important indicators of production efficiency. In line with previous reports on Pekin, Sansui Sheldrake, and layer ducks [8, 24, 25] and in broilers [26], weight gain and feed intake were unaffected by lowering dietary CP levels but did result in poorer FCR. Feng et al. [25] noted that dietary CP reduction from 18.04% to 13.4% in Sansui Sheldrake ducks, while maintaining constant amino acid profiles, resulted in lower weight gain (20.4-18.3 gm) and higher FCR (5.06 to 5.59) compared to the present study; this might be due to dietary CP levels and genotype differences.

Similar to the present study, Zeng et al. [12] reported that dietary protein levels from 15% to 17% influenced body weight, carcass weight, and breast meat in growing ducks. According to reports of Xie et al. [8], Sun et al. [9], and Wen et al. [13], dietary CP levels had no effects on carcass yield, breast meat, or leg meat yield in Sansui Sheldrake ducks, Pekin ducks, and quails, which supports the present findings. Similarly, Feng et al. [25] found non-significant differences in carcass, breast, or leg meat yield in Sansui Sheldrake ducks fed rations having 13.84% to 18.04% CP, although significant differences were found in abdominal fat deposition and are agreed upon partially with the current study. However, the impact of CP levels did not have any effect on breast meat yield, probably due to the ME:CP ratio, a balanced diet of amino acids, not just a balanced diet of total protein. The DP% (73.65 to 75.52) varied insignificantly among the four treatment groups in this study, which contained 16.5% to 21.0% CP. However, Xie et al. [8] obtained an almost similar DP% (71.40 to 74.70), providing an even lower CP-level diet that ranges from 13.54 to 17.22%.

The most important factor for both producers and customers is the quality of the meat. Kuźniacka et al. [27] found four different dietary protein sources did not significantly change ducks’ meat yield across the treatments for WHC and DL parameters in breast muscle and are comparable to the present study. However, Kokoszyński et al. [28] reported that CL had a significant effect on the breast muscle of Pekin ducks but not on leg muscle, which is comparable to our studies. Chartrin et al. [29] found that the CL value for breast muscles was 16.9% in Pekin ducks fed *ad libitum* and is comparatively lower than the present result. Furthermore, Oh et al. [30] reported that the CL values of the breast muscles of Pekin ducks ranged between 31.59 and 33.12%, which is similar to those reported here. Overall, meat quality parameters are influenced by estimating methods, age of the birds, and composition of the ration. Notably, there remains a lack of published data regarding meat quality parameters, particularly for crossbred ducks under varying CP levels.

Meat color characteristics, particularly lightness (L*), were influenced by dietary CP levels in TM of crossbred duck. Consumers generally prefer duck meat with balanced yellowness (b*) and moderate lightness (L*) because extremely dark muscle indicates less freshness, whereas extremely pale meat shows a poor ability to retain water. Similarly, while moderate values indicate freshness and high-quality food, extreme yellowness may be related to fat deposition [31]. Wang et al. [20] found significant differences in breast muscle yellowness (b) in Pekin ducks fed 13.5-17.5% CP that contradict the present study and might be due to dietary formulation and genotype. Kuźniacka et al. [27] reported significant differences in lightness (L*) and redness (a), with values varying from 36.02 to 44.02 and 12.09 to 17.39, respectively, in Pekin ducks, which is comparable to the current findings. However, Zhao et al. [19] reported that neither energy nor protein levels significantly affected meat color, contrasting with the present findings, likely because of genotype and diet composition. Altogether, meat color is affected by diet, freezing temperature, pH, myoglobin content, packaging, and oxygen exposure.

Hematological parameters such as Hb, PCV, and TEC are useful indicators of bird health and physiological status because they provide critical information about the bird’s ability to carry oxygen, level of hydration, and nutritional condition. According to Kavitha et al. [32] for White Pekin and indigenous ducks, the Hb values (9.71 ± 0.15 and 10.39 ± 0.14 gm/dl), PCV values (44.20 ± 0.47 and 50.00 ± 0.69%), and TEC values (2.20 ± 0.04 and 2.62 ± 0.03) × 10⁶/mm³ are almost similar to our findings. According to another study by Kumar et al. [33], the Hb and PCV of Arni ducks fed different CP and ME-containing diets ranged from 9.83 to 10.50 gm/dl and 45.83 to 48.33%, respectively. These values were marginally lower and slightly higher than our results. In addition, the ESR values of the local ducks and drakes of Southeastern Nigeria were 1.63 ± 0.35 and 1.95 ± 0.30, respectively, when feeding on white lupin seeds [34], which is nearly similar to our values. However, literature on the hematological profile of crossbred ducks remains scarce.

## 5. Conclusions

The present study indicated that dietary CP levels significantly influenced the growth performance, carcass yield, and meat quality parameters of BAU Black and White crossbred ducks. The diet containing 19.50% (T_3_) CP provided the favorable outcomes in terms of BWT, ADG, and FCR, indicating its suitability for optimizing growth performance. Moreover, meat quality traits such as DL and CL in breast muscle, as well as lightness (L*) of TM, indicated significant variation with dietary CP levels. Overall, a dietary CP level of 19.50% can be recommended for BAU Black and White Ducks to achieve optimal growth performance and feed efficiency without compromising carcass yield or meat quality.

## Data Availability

The data presented in this study are available from the corresponding author upon reasonable request.

## List of abbreviations

ADG, average daily gain; FCR, feed conversion ratio; BWT, body weight; CWT, carcass weight; DP, dressing percentage; BHWT, back-half weight; BMWT, breast meat weight; TWD, thigh with drumstick; WWT, wing weight; NWT, neck weight; GWT, giblet weight; HWT, head weight; DL, drip loss; CL, cooking loss; WHC, water holding capacity; L*, lightness; a, redness; b, yellowness; BM, breast meat; TM, thigh meat; Hb, hemoglobin; TEC, total erythrocyte count; ESR, erythrocyte sedimentation rate; PCV, packed cell volume; min, minutes; h, hours; gm, grams; gm/dl, grams per deciliter; ml, milliliters.
